# Rituximab-specific DNA aptamers are able to selectively recognize heat-treated antibodies

**DOI:** 10.1371/journal.pone.0241560

**Published:** 2020-11-05

**Authors:** Michael Kohlberger, Sabrina Wildner, Christof Regl, Christian G. Huber, Gabriele Gadermaier

**Affiliations:** 1 Department of Biosciences, Paris-Lodron University of Salzburg, Salzburg, Austria; 2 Christian Doppler Laboratory for Innovative Tools for Biosimilar Characterization, Paris-Lodron University of Salzburg, Salzburg, Austria; Lehman College, UNITED STATES

## Abstract

The monoclonal anti-CD20 IgG1 antibody rituximab is used as a first-line treatment for B cell lymphoma. Like all therapeutic antibodies, it is a complex protein for which both safety and efficacy heavily depend on the integrity of its three-dimensional structure. Aptamers, short oligonucleotides with a distinct fold, can be used to detect minor modifications or structural variations of a molecule or protein. To detect antibody molecules in a fold state occurring prior to protein precipitation, we generated DNA aptamers that were selected for extensively heat-treated rituximab. Using the magnetic bead-based systematic evolution of ligands by exponential enrichment (SELEX), we obtained six DNA aptamer sequences (40-mers) specific for 80°C heat-treated rituximab. *In silico* fold prediction and circular dichroism analysis revealed a G-quadruplex structure for one aptamer, while all others exhibited a B-DNA helix. Binding affinities ranging from 8.8–86.7 nM were determined by an enzyme-linked apta-sorbent assay (ELASA). Aptamers additionally detected structural changes in rituximab treated for 5 min at 70°C, although with lower binding activity. Notably, none of the aptamers recognized rituximab in its native state nor did they detect the antibody after it was exposed to lower temperatures or different physical stressors. Aptamers also reacted with the therapeutic antibody adalimumab incubated at 80°C suggesting similar aptamer binding motifs located on extensively heat-treated IgG1 antibodies. Within this work, we obtained the first aptamer panel, which is specific for an antibody fold state specifically present prior to protein aggregation. This study demonstrates the potential of aptamer selection for specific stress-based protein variants, which has potential impact for quality control of biopharmaceuticals.

## Introduction

Biopharmaceuticals are therapeutic agents which are produced in living cells or organisms and thus require tightly controlled production processes and product characterization [1–3]. Especially complex molecules like monoclonal antibodies (mAbs), produced in eukaryotic cell lines, depend on an orchestrated subunit assembly and distinct post-translational modifications [4]. The function and efficacy of therapeutic mAbs depends on their correct three-dimensional structure, and even minor variations might lead to impaired treatment efficacy or harmful side effects for patients [5]. During thermal denaturation, separate antibody subunits exhibit different levels of stability, while the exact unfolding mechanisms are not yet fully elucidated [6]. Rituximab, a monoclonal chimeric mouse/human anti-CD20 IgG1 antibody used for treatment of B cell lymphoma, was shown to unfold in three distinct steps [7, 8]. At 70°C, the CH2 region unfolds, followed by the Fab-domain at 75°C. The CH3 region only unfolds at 84°C, making it the most heat-stable part of the molecule. It was demonstrated that the completion of an initial oligomerization step subsequently leads to the formation of large aggregates and precipitation [7].

One straightforward option to selectively detect small molecules and ions, proteins or even cells is the aptamer technology. Aptamers are short single-stranded DNA or RNA molecules with a distinct fold and are obtained by a process termed systematic evolution of ligands by exponential enrichment (SELEX) [9–11]. Aptamers were shown to be highly specific and able to discriminate molecules with a difference of only one methyl group [12–15]. We recently generated a panel of DNA aptamers specific for rituximab [16]. Those aptamers were selected against native rituximab and can be used for biopharmaceutical quality control as they show altered recognition of thermally and physical stressed antibodies. So far, only a limited number of aptamers have been documented to be able to specifically detect changes in a protein’s conformation. These aptamers typically reveal changes in signal intensities and thus recognize deviations of unknown kind when compared to the native protein [16–18]. However, none of these aptamers is able to exclusively recognize an alternative surface structure of a heat-treated protein.

To detect antibody molecules in a fold state occurring immediately prior to protein precipitation, we generated DNA aptamers specific for thermally treated rituximab. For this purpose, rituximab immobilized to protein A was subjected to heat treatment of 80°C during the SELEX process. Using this setup, rituximab presented a favourable orientation and stabilized the Fc part when bound to protein A while the remaining molecule could undergo conformational changes. We obtained a panel of six high-affinity aptamers that selectively recognize extensively heat-treated rituximab, while they do not bind to the native molecule or physically stressed antibodies. These aptamers are suitable to act as pre-sensor for protein precipitation, which may be useful for studying the unfolding process of antibodies and could be applied in quality control of biopharmaceuticals.

## Material and methods

### Immobilization of rituximab on protein A magnetic beads

For target immobilization, 100 μl (2.7 x 10^8^ beads) of protein A magnetic beads (Dynabeads® Protein A, Life Technologies AS, Oslo, Norway) were used in conjunction with a magnetic stand. After washing the beads with 500 μl phosphate buffered saline, 0.02% v/v Tween 20 (PBST), 50 μg of rituximab (10 mg/ml, MabThera lot N7075B10, Roche, Basel, Switzerland), diluted in 400 μl 1 x PBST, were added. Rituximab and protein A were incubated with gentle rotation for 4 h at room temperature. After removal of the supernatant, the beads were washed 3 times with 500 μl PBST and stored at 4°C. Protein coating was verified by denaturing sodium dodecyl sulphate-polyacrylamide gel electrophoresis using 15% separating gels.

### *In vitro* selection of DNA aptamers using FluMag-SELEX

The aim of this SELEX procedure was the generation of aptamers specific for heat-treated rituximab. Therefore, we consequently re-used the material with protein A-immobilized rituximab that underwent heat-treatment at 80°C during each elution step. For *in vitr*o selection, a random unlabelled single-stranded DNA (ssDNA) library was used (IBA GmbH, Göttingen, Germany). The library consisted of randomized ssDNA strands of 40 nucleotides which were flanked by 18 fixed nucleotides required for PCR amplification (5´-ATACCAGCTTATTCAATT-N_40_-AGATAGTAAGTGCAATCT-3´). The corresponding primers used for PCR amplification and cloning were forward primer: 5´-ATACCAGCTTATTCAATT-3´, reverse primer: 5´-AGATTGCACTTACTATCT-3´, biotin-labelled reverse primer: 5´-biotin-AGATTGCACTTACTATCT-3´. Before each selection round, 80 μl of rituximab-immobilized protein A magnetic beads were washed eight times with 20 mM Tris pH 7.6, 100 mM NaCl, 2 mM MgCl_2_, 5 mM KCl, 1 mM CaCl_2_, 0.02% v/v Tween 20 (binding buffer). For the first selection round, 2 nmol of the ssDNA library were folded in 500 μl binding buffer at 94°C for 8 min, immediately cooled on ice for 15 min and kept at room temperature for 10 min. The folded aptamer library was incubated with the rituximab-coated magnetic beads for 1 h at RT with gentle shaking. The supernatant was removed and the beads were washed five times using binding buffer. Bound DNA was eluted by adding three times 200 μl of 40 mM Tris HCl pH 8.0, 10 mM EDTA, 3.5 M urea, 0.02% v/v Tween 20 (elution buffer) and incubation at 80°C for 8 min. The eluted DNA was precipitated by adding 60 μl (1/10 volume) of 3 M sodium acetate pH 5.2 and 1.65 ml (2.5 volumes) pre-cooled ethanol. One fifth of the eluted ssDNA was amplified via 20 parallel PCR-reactions, 100 μl each, consisting of 1x colorless GoTaq reaction buffer, 0.2 mM dNTP (Promega, Madison, USA), 0.5 μM forward primer, 0.5 μM biotin-labelled reverse primer and 3 U GoTag® DNA polymerase (Promega). The PCR settings were 94°C for 5 min, 25 cycles of 94°C for 30 s, 50°C for 30 s, 72°C for 30 s, followed by 72°C for 10 min. The DNA-strands were then separated by first coupling the double-stranded PCR products to streptavidin magnetic beads (Dynabeads® M-280 Streptavidin, Life Technologies AS, Oslo, Norway) via the biotinylated reverse primer, and then breaking the hydrogen bonds between the two strands using a 0.15 M sodium hydroxide solution [19]. By immobilizing the beads with the reverse strands coupled to them, the now uncoupled forward strands could be extracted and were used for the next round of selection, together with the rituximab-coated beads, which were re-used. A negative selection against magnetic protein A beads without rituximab was performed in round 6.

### Aptamer cloning and sequencing

The ssDNA eluted from the rituximab-immobilized beads in cycle 7 was amplified using the unmodified forward and reverse primers and GoTaq® DNA Polymerase (Promega, Madison, USA) as described above. The PCR product was ligated into a pGEM®-T easy vector (Promega) and transformed into NovaBlue *E*. *coli* cells. Of the resulting clones, 66 were selected for plasmid preparation using an EZ-10 spin column plasmid DNA Miniprep Kit (Bio Basic Inc., NY, USA). The plasmids containing the aptamers were then sequenced (Eurofins Genomics, Ebersberg, Germany), and 46 sequences with correct primer binding regions and a length of 40 bases in the variable region were analysed and aligned with Clustal Omega [20]. Finally, six selected aptamer sequences devoid of primer binding regions were synthesized as 5´-biotin-labelled oligonucleotides (Eurofins Genomics).

### Secondary structure prediction and circular dichroism spectroscopy of aptamers

*In silico* prediction of the secondary structure of aptamers was performed by free energy minimization algorithm using the online tool “mfold” (http://unafold.rna.albany.edu) [21]. The settings were linear DNA sequence, 22°C folding temperature, ionic conditions: 100 mM Na^+^ and 2 mM Mg^2+^ with correction type: oligomer. For the remaining settings, the suggested default values were used. To calculate the G-C content of aptamer sequences, the online tool “DNA calculator” was used (http://www.endmemo.com/bio/gc.php), for G-score calculation, the online tool “QGRS Mapper” was utilized (http://bioinformatics.ramapo.edu/QGRS/analyze.php), with default settings. Circular dichroism (CD) spectra were measured utilizing a JASCO J-815 spectropolarimeter (Jasco, Tokyo, Japan). For this purpose, aptamers (without biotin) were dissolved in the binding buffer (20 mM Tris pH 7.6, 100 mM NaCl, 2 mM MgCl_2_, 5 mM KCl, 1 mM CaCl_2_, 0.02% v/v Tween 20) and folded at 4 μM according to the SELEX protocol provided above. Recording of UV-spectra ranging from 200–320 nm was done at 20°C and results are displayed as mean residue molar ellipticity. For further G-quadruplex analysis, singular value decomposition was conducted using an R-script developed by del Villar-Guerra et al. [22].

### Thermal and physical treatment of rituximab

Aliquots of rituximab (MabThera, lot N7075B10) were treated in different ways in order to analyse the aptamer binding behaviour. For all treatments, 50 μl aliquots of rituximab solution were prepared in 1.5 ml vials. For heat treatment, the vials were incubated in a heating block either at 60°C for 5 min, 70°C for 5 min, 70°C for 1 min, 75°C for 1 min or 80°C for 1 min, respectively. The protein aliquots were centrifuged at 14000 rpm for 5 min and the supernatant used for further analyses. For UV-treatment, the vials were kept on a standard benchtop trans-illuminator (15 W, 312/302 nm) at 100% setting for 10 min. For freeze-thaw-vortex, the aliquots were incubated at -20°C until thoroughly frozen, then thawed at room temperature, and then vortexed for 30 sec at the highest setting. This process was repeated for a total of 10 times. For lyophilisation, the aliquots were lyophilised using a CentriVap with ColdTrap (Labconco, Kansas City, US), and then reconstituted in an original volume of ultrapure water.

### Enzyme-linked apta-sorbent assay (ELASA)

White Maxisorp Fluoronunc plates (Thermo Fisher Scientific Nunc A/S, Roskilde, Denmark) were coated with Thermo Scientific^TM^ Pierce^TM^ recombinant protein A at a concentration of 4 μg/ml in 0.2 M ammonium bicarbonate buffer pH 9.4 for 3 h at RT. Plates were then washed with 137 mM NaCl, 2.7 mM KCl, 10 mM sodium phosphate pH 7.4, 0.05% v/v Tween 20 (PBST) and blocked with PBST, 0.5% w/v BSA for 2 h at room temperature. Then, the plates were incubated overnight at 4°C with native or heat-treated rituximab, adalimumab or trastuzumab, diluted to 4 μg/ml in PBST. The plates were washed with 137 mM NaCl, 2.7 mM KCl, 10 mM Tris, pH 7.4, 0.05% v/v Tween 20 (TBST). Biotinylated aptamers at a concentration of 4 μM in binding buffer (20 mM Tris pH 7.6, 100 mM NaCl, 2 mM MgCl_2_, 5 mM KCl, 1 mM CaCl_2_, 0.02% v/v Tween 20) were heated to 94°C for 8 min, cooled on ice for 15 min and kept at RT for 10 min. Folded aptamers were diluted to a concentration ranging from 4–500 nM in dilution buffer, and incubated at room temperature protected from light for 2 h. After washing with TBST, bound aptamers were detected with horseradish peroxidase-conjugated streptavidin (Caltag Laboratories, Carlsbad, CA, USA). BM Chemiluminescence ELISA Substrate (Roche, Mannheim, Germany) was used as substrate and luminescence was measured with an Infinite M200 PRO plate reader (Tecan Group Ltd, Männedorf, Switzerland).

### Mass spectrometry of heat-treated rituximab

Three aliquots of rituximab solution, 50 μl each, were kept for 1 min at 80°C, 5 minutes at 70°C or 5 minutes at RT respectively. Subsequently, all samples were subjected to disulfide bond reduction, alkylation and tryptic digestion. Chromatographic separation of 1 μg digested rituximab was carried out on a Dionex™ UltiMate™ 3000 Rapid Separation system, after which mass spectrometry was conducted on a Thermo Scientific^TM^ Q Exactive^TM^ benchtop quadrupole-Orbitrap mass spectrometer equipped with a Thermo Scientific^TM^ Ion Max^TM^ ion source housing with a heated electrospray ionization (HESI) probe. Data acquisition was conducted using Thermo Scientific^TM^ Chromeleon 7.2 Chromatography Data System (CDS). Data analysis of mass spectra and peak assignment was performed using Thermo Scientific^TM^ BioPharma Finder software 3.0. Further details concerning the mass spectrometry method can be found in the S1 File.

### Circular dichroism analysis and dynamic light scattering of native and heat-treated rituximab

For analysis of secondary structure elements, circular dichroism (CD) spectra of native and heat-treated (incubated at 70°C for 5 min, and 80°C for 1 min) samples of rituximab were analysed using a Jasco J-810 spectropolarimeter (Jasco, Tokyo, Japan). After brief centrifugation, the proteins were diluted to 0.05 mg/ml in 100 mM sodium phosphate buffer pH 7.0. Far UV-spectra (190–260 nm) were recorded at 20°C in the CD cuvette and results are presented as mean residue molar ellipticity. The aggregation status of native and heat-treated samples (incubated at 70°C for 5 min, and 80°C for 1 min) of rituximab was analysed by dynamic light scattering (DLS) (Viscotek 802, Houston, TX, USA). The proteins were diluted to a protein concentration of 0.1 mg/ml in 10 mM sodium phosphate buffer pH 7.0 and measured using a quartz cuvette. Data were accumulated for 10 x 5 s and the correlation function was fitted into the combined data curve from which the intensity distribution was calculated. The hydrodynamic radius and estimated molecular mass of the proteins were calculated with the provided software.

### Aptamer binding analysis to different monoclonal IgG1

For these analyses, the ELASA assay conditions were kept as described above. In addition to rituximab, the IgG1 monoclonal antibodies adalimumab (Humira, AbbVie Ltd., North Chicago, US) and trastuzumab (Herceptin, Roche, Basel, Switzerland) were used for the experiment. Both antibody solutions were diluted to the original concentration of rituximab (10 mg/ml) with ultrapure water immediately before the heat treatment.

### Statistical analyses

Data analyses were performed in GraphPad Prism 7.03. A non-linear regression (one site—total and nonspecific binding) was used for Kd determination. A non-parametric test was performed, comparing all samples to each other, with corrections for multiple comparisons according to Dunn. A p-value <0.05 was considered significant.

## Results

### *In vitro* selection of DNA aptamers against heat-treated rituximab

To obtain DNA aptamers against the heat-treated monoclonal IgG1 antibody rituximab, a DNA-library consisting of 10^15^ different single-strand oligonucleotides with a random part of 40 bp was used as template for initial selection. In total, six aptamer selection cycles and one counter cycle were performed to enrich DNA sequences with specificity to rituximab treated at 80°C during the aptamer elution process. After cloning and transforming the DNA of the enriched SELEX cycle seven, 46 plasmids were sequenced resulting in 31 different sequences. Aptamer sequences which were identified at least twice (C2, C4, C5, C7, C10, C57) were selected and showed a G-C content of 45–50% (Table 1). Using the FluMag-SELEX technique, we obtained six distinct DNA aptamer sequences for subsequent structural and immunological characterization.

**Table 1:** Aptamer sequences obtained after SELEX using heat-treated rituximab.

aptamer	sequence	frequency	G-score	G-C content (%)
C2	TAGTATGGACTTGTCTTCTTGTCAGAGTCTGGCAGCACGT	3/46	-	47.5
C4	GGCACTTGTTTGCTCGAACGATGGTTTGTGCTTTTGTGTT	2/46	-	45
C5	CGCTCCGTTTACTCGGTCTGCTATTGGCTTGCCTTTGTTT	6/46	-	50
C7	**GG**CCATTGT**GG**ACTTCTTT**GG**GTAATTCA**GG**GGCTCGATT	5/46	30	50
C10	ACTTCGGCTAGTTAGGGGGTAGTTTAGATCGTCTCTACAT	2/46	-	45
C57	GGCGTATGCAGTTTTGTAGGGTTATCAAGGCTTGACGATT	3/46	-	45

Highlighted in bold and underlined are guanine bases (G) predicted to be involved in the formation of G-quadruplex structures. The G-score value represents the probability of quadruplex formation by G-rich sequences (maximum value obtained with default settings is 105).

### *In silico* fold prediction of selected aptamers

**Fig 1:**
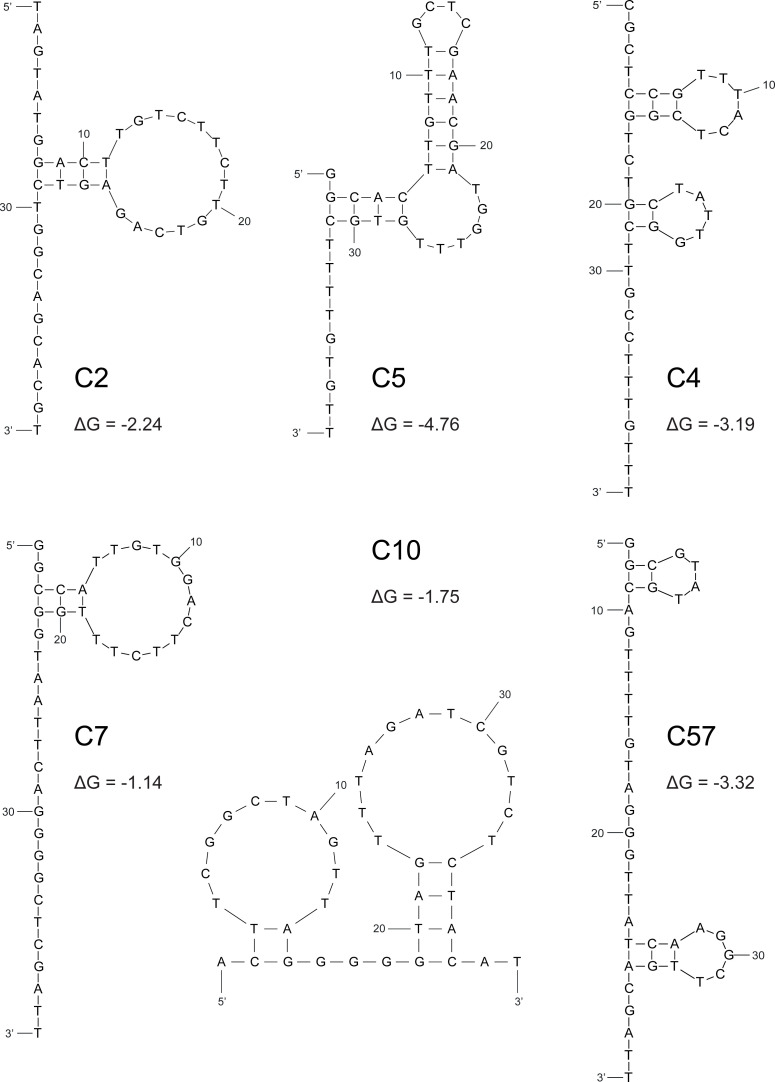
*In silico* aptamer structure prediction. Schematic depiction of secondary structure prediction and negative free energies (ΔG) of folding as computed by the online tool “mfold”.

To predict the secondary structure of aptamer sequences *in silico*, we used the online analysis tool “mfold”. For each range of possible structures, the ones with the lowest free energy (ΔG) were selected and are presented in Fig 1. Structural predictions revealed both hairpin formations of varying lengths and loops of different sizes for all aptamers. Lowest ΔG were observed for aptamers C5 (ΔG = -4.76), C57 (ΔG = -3.32) and C4 (ΔG = -3.19). In addition, the possibility of G-quadruplex formation was analysed via the online tool “QGRS Mapper”. Of note, aptamer C7 showed a G-score of 30 and presents four stretches of two guanine bases each that could be involved in G-quadruplex formation (Table 1).

### Aptamers fold as B-DNA helix or quadruplex structure

**Fig 2:**
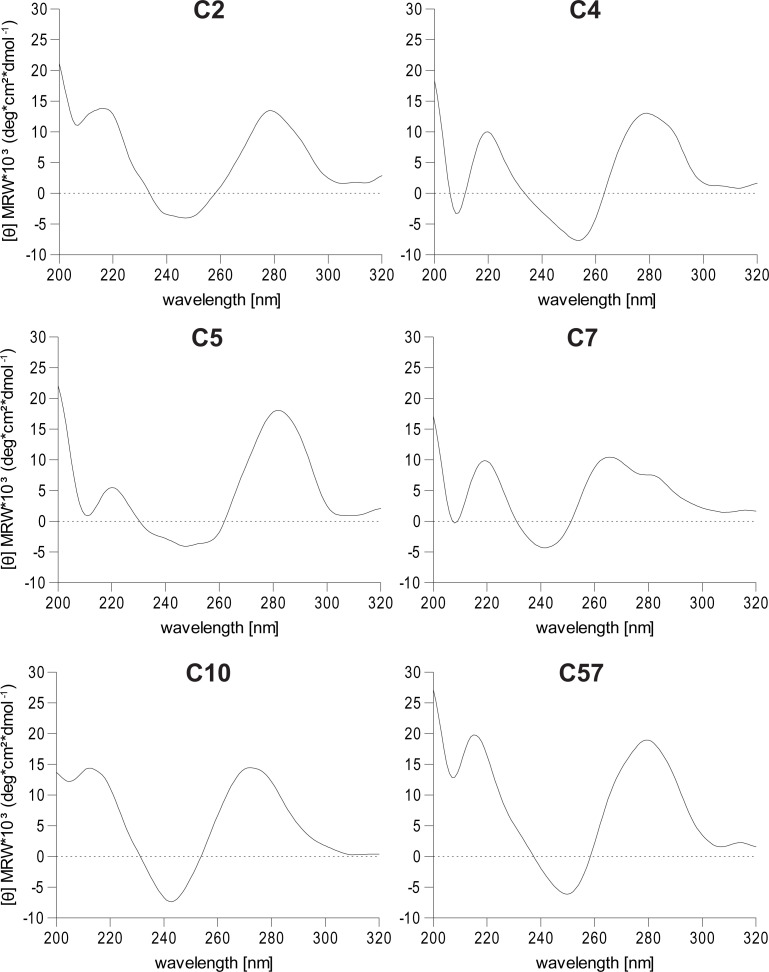
Aptamer secondary structure analysis using circular dichroism spectroscopy. Circular dichroism spectra of non-biotinylated, folded aptamers in binding buffer, measured at 20°C.

To experimentally verify the secondary structure of aptamers, circular dichroism spectroscopy was employed. For this purpose, non-biotinylated aptamers were folded in aptamer binding buffer analogously to the protocol used during SELEX process. All circular dichroism spectra, except for aptamer C7, were similar in their amplitude position (Fig 2). The negative minima around 245 nm and positive maxima around 280 nm indicates the presence of a B-DNA helix structure [23]. For C7, analysis via singular value decomposition revealed a mixture of G-quadruplex structures composed of 36% parallel, 22% antiparallel and 41% hybrid conformations [22]. Using circular dichroism, we could experimentally confirm the G-quadruplex structure of C7 and a B-DNA helix fold for all the remaining aptamers.

### Aptamer titration for recognition of heat-treated rituximab

**Fig 3:**
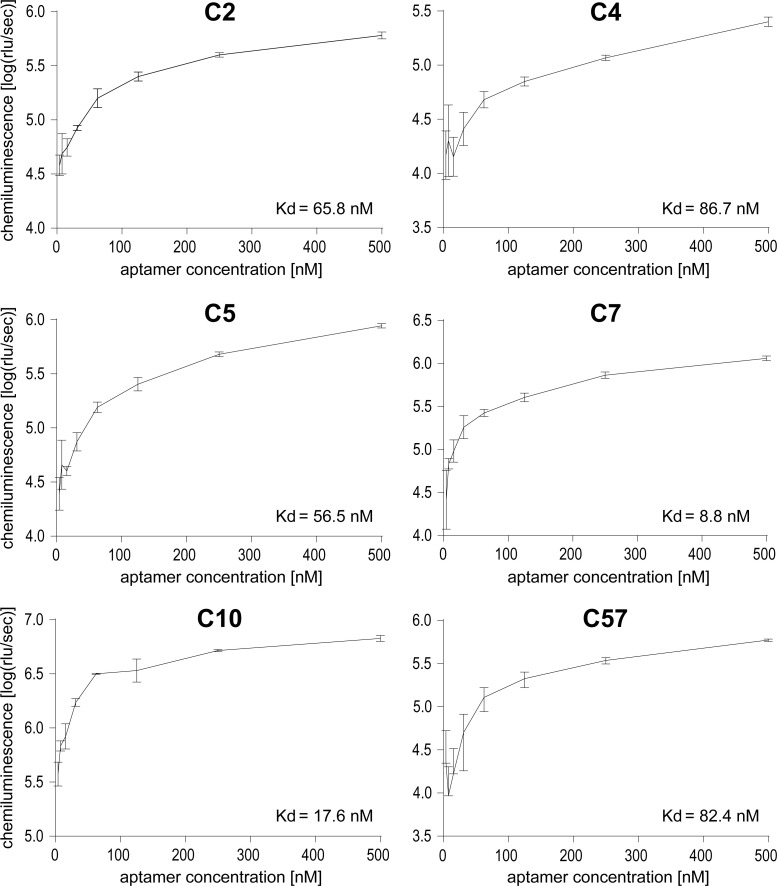
Titration of aptamers for heat-treated rituximab binding using ELASA. Rituximab was incubated at 80°C for 1 minute and bound to immobilized protein A. Aptamers were applied in working concentrations ranging from 4–500 nM. Mean chemiluminescence and standard deviations of three replicates per aptamer dilution are presented.

Selected aptamer sequences without primer regions were obtained as 5´-biotinylated oligonucleotides and tested in an ELASA. For this purpose, ELASA plates were coated with protein A to subsequently bind heat-treated rituximab. To determine the optimal concentration for aptamer binding, titration experiments were conducted with rituximab incubated at 80°C for 1 min and aptamer concentrations ranging from 4–500 nM. The incubation at 80°C was chosen to emulate the treatment that rituximab underwent during the SELEX process. The time span of 1 min was the longest possible exposure time at this temperature before rituximab would visibly precipitate while in the original concentration and buffer. Highest affinities was determined for aptamers C7 and C10 presenting Kd values of 8.8 nM and 17.6 nM, respectively. Using the streptavidin-HRP complex in conjunction with a chemiluminescence substrate, an aptamer concentration of 250 nM provided robust detection for all aptamers and was used for subsequent analyses (Fig 3).

### Aptamers selectively recognize extensively heat-treated rituximab

**Fig 4:**
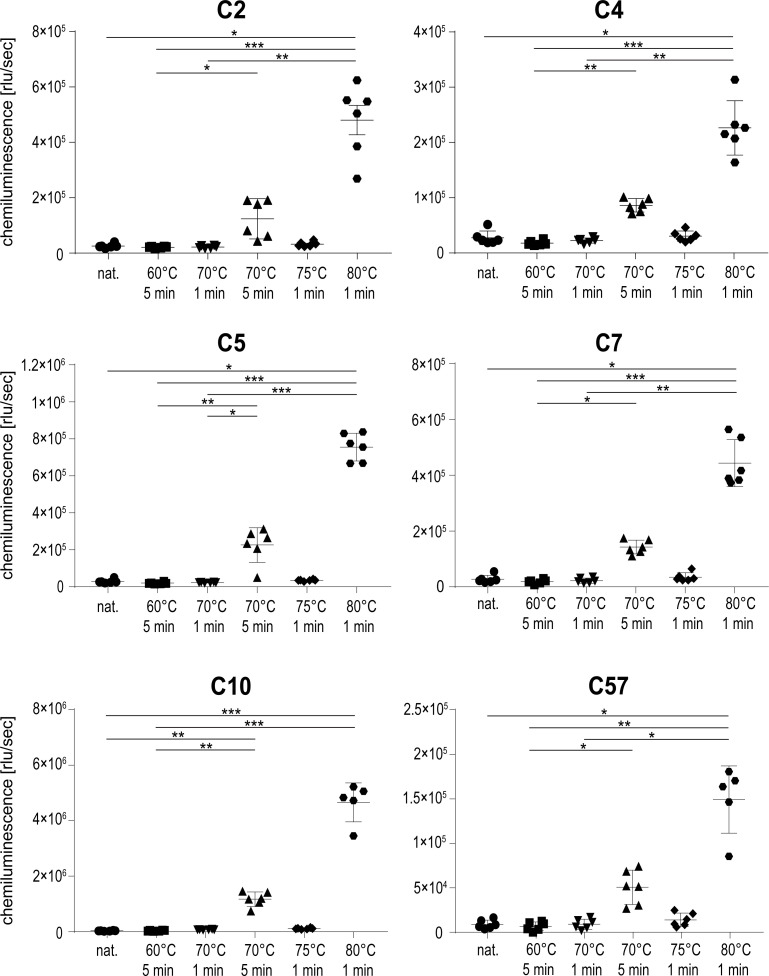
Aptamer binding to native and differentially heat-treated rituximab using ELASA. Shown are the means and standard deviations of six replicates per sample and aptamer. One replicate value had to be omitted from sample 80°C 1 min for aptamer C10 and C57 respectively, because they were disproportionately low and likely inconclusive for the measurement. A non-parametric test was performed, comparing all samples to each other, with corrections for multiple comparisons according to Dunn (*, p ≤ 0.05; **, p ≤ 0.01; ***, p ≤ 0.001).

To investigate whether aptamers selected for recognition of heat-treated rituximab would recognize other states of the protein, native and differentially heat-treated rituximab was tested in ELASA. Rituximab samples were either left untreated or heated for 5 min at 60°C and 70°C, or for 1 min at 70°C, 75°C and 80°C. Heating at 70°C for 5 min and 80°C for 1 min again reflected the longest possible treatment without visible protein precipitation. As anticipated, all aptamers recognized rituximab treated at 80°C resembling the temperature used in the SELEX process (Fig 4). All aptamers also recognized rituximab treated for 5 min at 70°C, although with a 2.5–3.9 times lower binding efficiency. Interestingly, neither the native protein nor rituximab treated at any other temperature or time span were recognized by the aptamers. Aptamers therefore selectively recognize structural features found on extensively heat-treated rituximab which seem absent in the original protein conformation.

### Heat-treatment of rituximab had no influence on its secondary structure or post-translational modifications but triggered oligomerization

**Fig 5:**
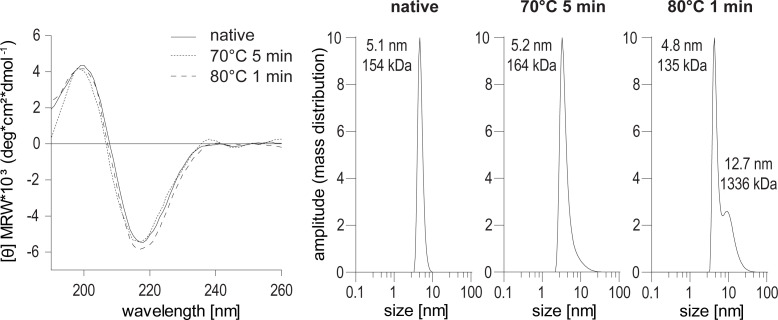
Secondary and tertiary structure of native and heat-treated rituximab. Circular dichroism spectra of native and thermally treated antibody in SELEX binding buffer (left panel). Dynamic light scattering of native and thermally treated rituximab (right panel). MRW, mean residue molar ellipticity.

To investigate the physico-chemical properties of rituximab upon treatment at high temperatures, we analysed its primary and secondary structure as well as the aggregation behaviour. As the heat-treated protein preparations showed a time-dependent susceptibility for oligomerization, all aliquots were briefly centrifuged immediately before measurements to remove precipitated proteins. Using mass spectrometry, the primary structure and post-translational modifications of native, 5 min 70° and 1 min 80°C treated samples were analysed and compared (S1 Fig). No relevant differences in glycation, succinimide formation or composition of the N-glycans at residue 301 were observed. Protein oxidation revealed comparable results except for a gradual increase of tryptophan oxidation at residue 106 located in the heavy chain of the antibody. The relative abundance however increased on average from 0.5% to 2.5% and the overall effect is thus comparably small. The circular dichroism spectra, displaying the protein’s secondary structure elements, were highly similar for native and heat-treated rituximab (Fig 5A). CD curves showed a minimum around 195 nm and a maximum around 200 nm and thus resemble proteins with predominant β-sheet content. To analyze the aggregation behavior, we additionally analyzed all samples by dynamic light scattering (DLS) (Fig 5B). Native rituximab was detected as one single sharp peak with a calculated hydrodynamic radius of 5.1 nm (~155 kDa), indicative of a monomeric molecule. The sample treated for 5 min at 70°C showed a very similar size of 5.2 nm, while the peak shape presented a slight slant along the bottom of the peak. Based on the software calculation, the protein is still in a monomeric conformation. The sample treated for 1 min at 80°C showed that 50.8% of the protein were monomers. In addition, a second peak with an increased hydrodynamic radius of 12.7 nm (~1337 kDa) indicates the presence of up to 49.2% rituximab oligomers in the protein preparation. Regarding the physico-chemical characteristics, a difference in the oligomerization state of rituximab was observed while neither the primary nor the secondary structure were significantly affected by the heat-treatment.

### Aptamers do not bind to UV- and physically stressed rituximab

**Fig 6:**
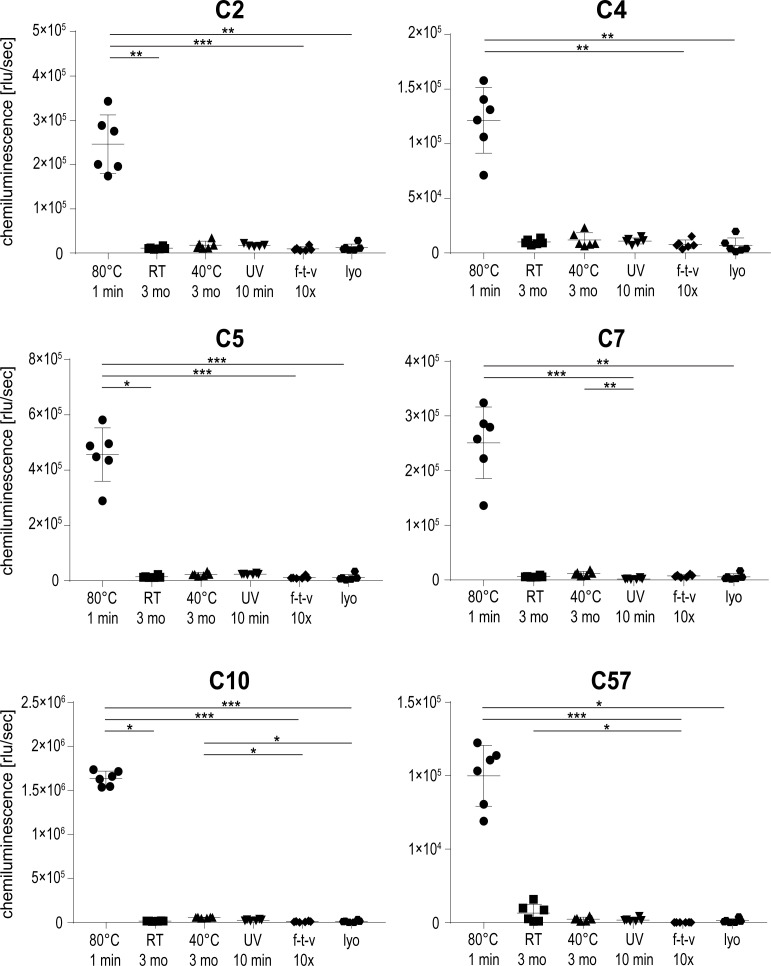
Aptamer binding behavior with differentially treated rituximab using ELASA. Shown are the means and standard deviations of six replicates per sample and aptamer. A non-parametric test was performed, comparing all samples to each other, with corrections for multiple comparisons according to Dunn (*, p ≤ 0.05; **, p ≤ 0.01; ***, p ≤ 0.001).

To investigate if aptamers would bind to rituximab stressed in other ways than short-term high temperature treatment, an ELASA using a variety of differentially treated rituximab samples was conducted. Two samples of rituximab underwent thermal incubation, with storage at room temperature and 40°C for 3 months each, respectively. For a UV treatment, an aliquot was kept on a UV benchtop trans-illuminator at 100% setting for 10 min. To simulate physical stress, one sample was repetitively subjected to a combination of freeze/thaw and shaking while another sample was lyophilized once and then re-suspended in the original amount of ultrapure water. As a positive control, a sample of rituximab treated at 80°C for 1 min was used. While all aptamers reacted to the positive control, none of the rituximab samples treated at lower temperatures or other stress conditions showed aptamer binding (Fig 6). Thus, selected aptamers prove to be highly specific for rituximab stressed at high temperatures.

### Aptamer binding behaviour with other therapeutic IgG1 antibodies

**Fig 7:**
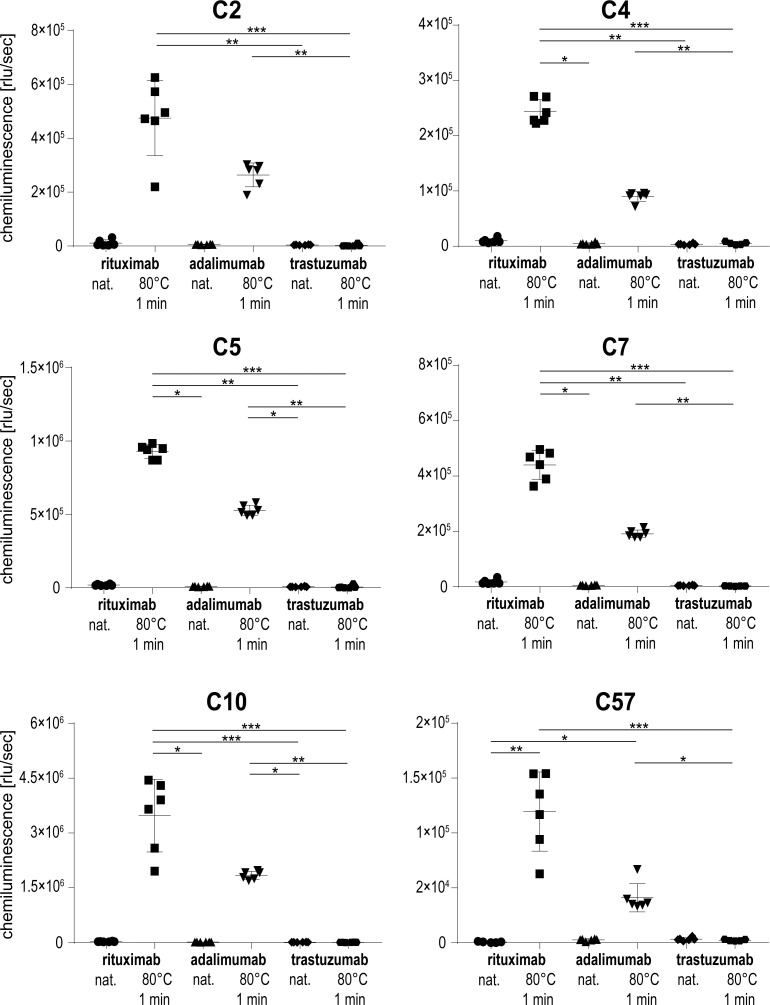
Aptamer binding behavior with other IgG1 antibodies. Shown are the means and standard deviations of six replicates per sample and aptamer. A non-parametric test was performed, comparing all samples to each other, with corrections for multiple comparisons according to Dunn (*, p ≤ 0.05; **, p ≤ 0.01; ***, p ≤ 0.001).

In order to test whether aptamers would bind to other IgG1 antibodies, two therapeutic monoclonal antibodies, i. e. the human adalimumab and the humanized trastuzumab were tested alongside the chimeric antibody rituximab. Prior to heat-treatment, all antibodies were diluted to the same concentration as rituximab to allow for treatment consistency during incubation for 1 min at 80°C. In addition to heat-treated rituximab, adalimumab was also recognized by the aptamers, albeit to a lower degree ranging from 34–57% (Fig 7). In contrast, no signal was detected for heat-treated trastuzumab and no reactivity to any of the mAbs in their native conformation was observed. These results indicate that selected aptamers are not only specific for heat-treated rituximab but have the potential to detect other heat-treated IgG1 antibodies as exemplified by adalimumab.

## Discussion

Aptamers are widely appreciated for their remarkable versatility and specific recognition of their selected target [12–14]. With regard to protein conformation, aptamers with different affinities for native and thermally treated or misfolded proteins have been generated [16–18]. As antibodies exhibit structurally complex subunits and comprise relevant biopharmaceuticals [24, 25], we selected the anti-CD20 monoclonal antibody rituximab as specific target. Within this study, we were able to generate aptamers that selectively recognize heat-induced structural surface motifs of rituximab which appear immediately prior to protein precipitation. To our knowledge, this is the first panel of aptamers that selectively dedects a heat-induced fold-state of multiple antibodies while the same proteins in their native conformations are not recognized.

In this study, we used the well-established FluMag-SELEX technique to screen for aptamers specific for heat-treated rituximab [26, 27]. Rituximab was immobilized onto protein A magnetic beads for two reasons, i) distinct Fab orientation of singular antibody molecules and ii) direct employment of consecutive 80°C treatments during the aptamer elution step in each SELEX cycle are possible. This setup takes the high stability of the CH3 domain into consideration [7] and suggests an additional stabilization of the CH2 domain when bound to protein A. A nucleotide library consisting of 40 bases in the random part was used for screening. This aptamer length is within the recommended range of 30–50 nucleotides, and provides an attractive balance between sequence and fold diversity as well as structural stability [28]. The number of cycles was chosen as a compromise between optimal sequence enrichment and minimal loss of unique sequences, according to the results of previous studies on aptamer dynamics during SELEX [16, 27, 29–31]. The counter-selection without rituximab after cycle 6 was performed to remove unspecific aptamers but on the other hand avoid the early loss of otherwise suitable candidates [27]. Out of the 46 retrieved sequences, aptamers C5 and C7 were identified within several clones, while others were obtained with a lower frequency suggesting a moderate selection pressure during the SELEX procedure. These occurrence numbers are however similar to our previous SELEX performed with native rituximab, which also demonstrated that sequences with low copy frequency were ultimately among the aptamers with highest affinity [16].

Using a bioinformatic nucleotide fold prediction tool, aptamers were shown to take on distinct conformations, containing both hairpin and loop formations. For aptamer C7, a low negative folding energy value was predicted, which taken together with its repetitive guanine-motifs (tetrads of GG bases) suggested a G-quadruplex structure [32, 33]. This could be experimentally verified by CD spectroscopy, where the spectrum of aptamer C7 indicated a mixture of quadruplex structures [22]. The CD spectra of the remaining aptamers suggested B-DNA-helical folds [34]. It is noteworthy to mention that all CD measurements were conducted in aptamer binding buffer, as the presence of cations is relevant for quadruplex formation [35].

To determine the affinity of the aptamers to heat-treated rituximab, different concentrations of biotinylated aptamers were tested in an ELASA [36, 37]. Prior to applying it to the ELISA plate, rituximab was incubated in the binding buffer with identical concentration at 80°C for 1 minute, to emulate the conditions applied to the protein during the SELEX process. In contrast to the SELEX, the incubation time had to be limited to 1 minute due to the fact that the protein was heat-treated in solution which causes rapid precipitation upon longer thermal exposure. Furthermore, the polystyrene-plates were pre-coated with protein A, to ensure a uniform orientation of the rituximab molecules and conditions close to SELEX. Most efficient aptamer binding was verified for C7 and C10. In comparison to aptamers previously obtained for native rituximab, the affinity of these aptamers is on average ten times higher, however the Kd was determined by two different methods [16]. While C7 was among those sequences obtained with higher frequency (5/46 clones), C10 was only sequenced twice which again highlights that the number of identical clones is not directly related to the binding strength [16].

To determine whether the aptamers were solely specific for rituximab heated at 80°C, an ELASA using immobilized rituximab treated at different temperatures was conducted. All aptamers recognized their original selection target, namely rituximab heated to 80°C. In addition, the protein treated for 5 min at 70°C was detectable to a lower degree, while all other protein preparations were not recognized. We therefore conclude that our aptamers exclusively recognize distinct surface structures, which are induced by thermally stressing rituximab. As initial analyses showed, our aptamers also exclusively recognize heat-treated rituximab in an ELASA-setup without protein A, ruling out the possibility that the aptamers bind to our immobilizing agent. Both the temperature as well as the incubation time seem crucial to obtain the specific conformation of the protein that is subsequently recognized by the aptamer. Specific detection of rituximab treated at 70°C required the heat-treatment to last for 5 min, which represents the longest time span immediately before the protein visibly precipitates. We thus suggest that our aptamers are able to sense a specific rituximab conformation that immediately precedes a well-documented phase of protein aggregation [7]. While aptamers can be generated against virtually any target including proteins with alternative folds, it is challenging to obtain specific antibodies recognizing unfolded structures of proteins. Monoclonal antibodies are typically generated by mouse immunization, however this requires an *in vivo* immune response which is typically not established against unfolded proteins. In this context, aptamers are an excellent *in vitro* method to select targets presenting an alternative fold.

To further investigate potential changes in the primary and secondary structure of rituximab during thermal treatment, we compared the native protein to thermally treated samples using mass spectrometry, circular dichroism and dynamic light scattering. Heat-treatment did not induce considerable changes in the abundance of post-translational modifications like glycosylation, oxidation or glycation. The only noticeable difference shows a different oxidation of heavy chain tryptophan at residue 106. However, as the relative abundance only increases from 0.5% to 2.5%, the effect is still comparably small and unlikely to explain the detection by all investigated aptamers.

Analysis of heat-treated rituximab by circular dichroism revealed very similar spectra and corresponding secondary structure elements as compared to native rituximab. It has to be noted that after thermal treatment, the protein aliquots started to precipitate and for measurement, the supernatant was used as solid particles cannot be measured by CD. In a previous study, the unfolding behaviour of heat-stressed rituximab showed that all antibody domains except for CH3 unfolded upon incubation at >80°C. In contrast to our work, longer incubation times and higher temperatures were employed which finally resulted in complete and irreversible protein precipitation which prevented refolding. Isolated antibody domains on the other hand possess the capacity to refold after heat denaturation [6]. It thus seems likely that our very short heat treatment at 80°C predominately causes local structural changes enabling refolding of the secondary structure elements. This resulted in CD curves and secondary structure elements similar to the native protein observable in the protein solution. On the other hand, a temperature- and time-dependent increase in the hydrodynamic radius of rituximab was observed in our study and by Andersen et al. [7]. DLS analyses revealed a tendency for oligomerization after 5 min at 70°C, while a mix of mono- and oligomers was observed after 1 min at 80°C. In line with the multi-domain aggregation study of antibodies, we conclude that prolonged heating at 80°C leads to further aggregation and finally precipitation of the antibody [6, 7]. It is noteworthy to mention that during aptamer screening rituximab was consequently bound to protein A, which prevented the formation of aggregates by keeping the protein molecules spatially separated [7]. We thus conclude that our newly generated aptamers can specifically detect heat-induced tertiary structural changes, which are not detectable by circular dichroism or mass spectrometry.

To determine whether other kinds of stress would cause a similar modification in rituximab, the antibody was exposed to physical stresses. None of the tested conditions like UV light, freezing/thawing or lyophilisation caused a modification that enabled the aptamers to bind to the protein surface. This finding underlines the high specificity of the aptamers, which react exclusively to protein treated in the same or a very similar way as their SELEX target. On the other hand, our aptamers were able to recognize the heat-treated therapeutic antibody adalimumab, although to a lesser degree. The amino acid sequence of adalimumab is entirely human-based while rituximab is a chimera of human Fc- and murine Fab-domains. We thus speculate that the binding region of the aptamers is on a similar motif in the three-dimensional structure of both antibodies, most likely located within the Fab constant or hinge region. Interestingly, no signal was obtained for the heat-treated humanized antibody trastuzumab, which most likely relates to the different heat-denaturation behaviour of this biopharmaceutical. While rituximab and adalimumab entirely precipitated when heated for longer than 1 min at 80°C, trastuzumab did not show signs of denaturation when treated even longer or at higher temperatures. This is most likely caused by the presence of the protein-stabilizing agent trehalose in the formulation buffer [38, 39].

Within this study, a panel of aptamers recognizing distinct structural determinants of extensively heat-treated antibodies were generated. The use of aptamers provides a unique opportunity to select for structurally modified proteins that are impossible to screen for when using *in vivo* generated antibodies. In a broader context, aptamers selected for unfavourable protein conformations might be used as pre-sensors for protein precipitation and misfolded proteins in biopharmaceuticals.

## Supporting information

S1 Fig Mass spectrometry analysis of native and heat-treated rituximab S1 File. The plots display the relative abundance of the respective amino acid modifications as determined by mass spectrometry. LC, light chain; HC, heavy chain.
(PDF)

S1 File Supporting materials and methods. (PDF)
