# Supplementary figures and images for: Rituximab-specific DNA aptamers are able to selectively recognize heat-treated antibodies
Source: PLoS One. 2020 Nov 5;15(11):e0241560. doi: 10.1371/journal.pone.0241560 (PMC7644011; doi:10.1371/journal.pone.0241560)

### glycation

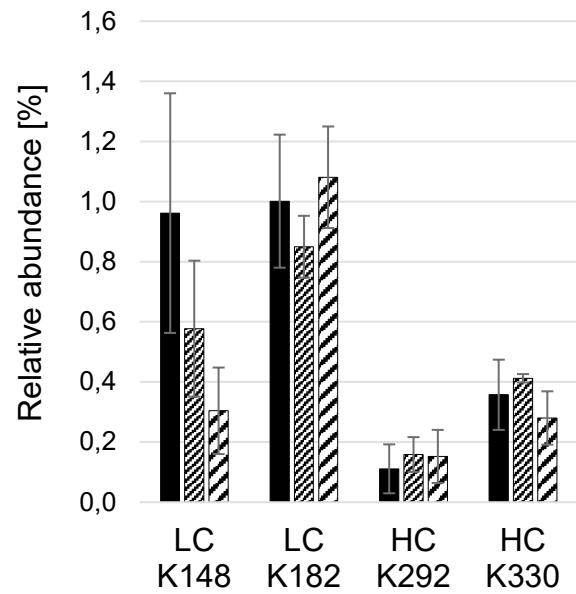

### succinimide

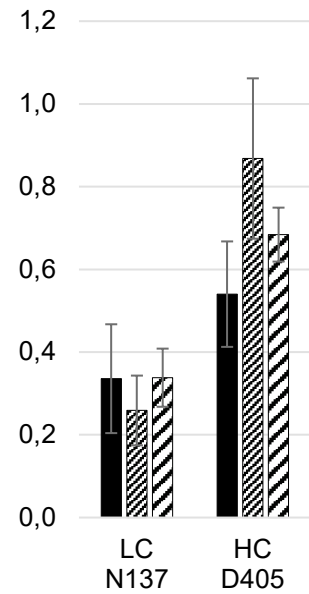

### oxidation

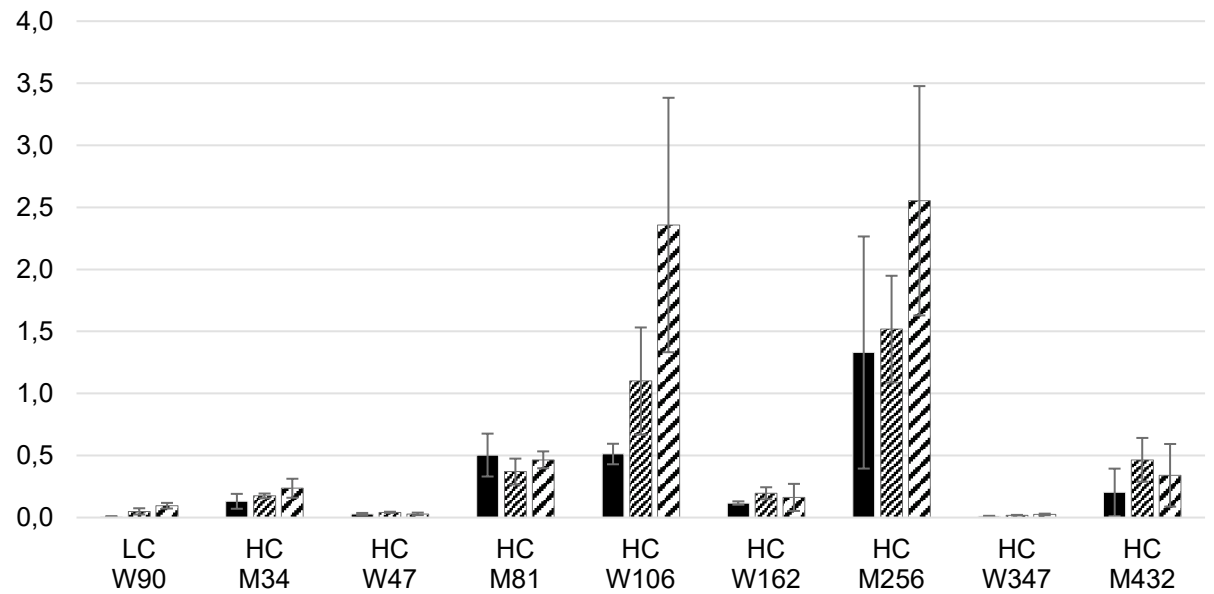

### N-glycosylation HC N301

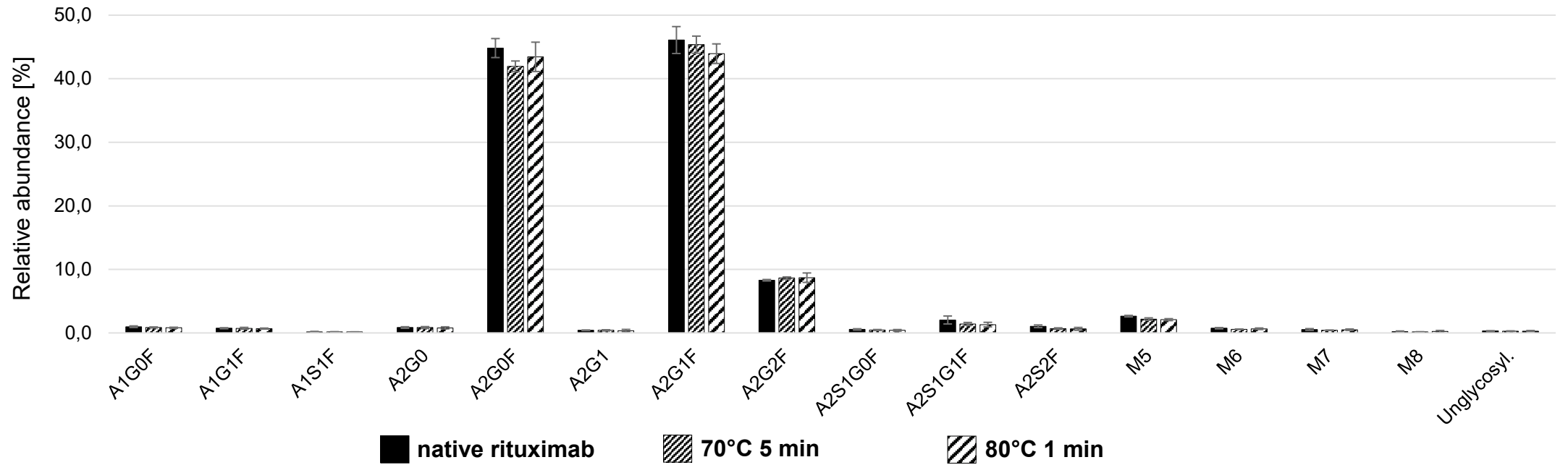

Supplement: S1 Fig — The plots display the relative abundance of the respective amino acid modifications as determined by mass spectrometry. LC, light chain; HC, heavy chain. (PDF) [file pone.0241560.s001.pdf]
