# Supplementary material for: Rituximab-specific DNA aptamers are able to selectively recognize heat-treated antibodies
Source: PLoS One. 2020 Nov 5;15(11):e0241560. doi: 10.1371/journal.pone.0241560 (PMC7644011; doi:10.1371/journal.pone.0241560)
Supplement: S1 File — (PDF) [file pone.0241560.s002.pdf]

Supporting materials and methods

Sample preparation for mass spectrometry

Three biological replicates of rituximab á 50 µl were stressed for 1 min at 80°C, 5 min at 70°C. Subsequently the native and heat-stressed samples were diluted 1:1 (*v/v*) to a concentration of 5 mg/ml in 4 M guanidine hydrochloride and 5 mM tris(2-carboxyethyl)phosphine. Disulfides were reduced for 15 min at 37 °C and subsequently alkylated by addition of 20 mM iodoacetamide and incubation for 30 min at 22°C. Subsequently the samples were buffer-exchanged to 175 mM ammonium acetate using Amicon Ultra 0.5 ml centrifugal filters (3 kDa cut-off, Sigma-Aldrich) according to the manufacturer's protocol. Proteolysis was performed via addition of Trypsin-Lys-C mixture at a mAb to enzyme ratio of 50:1 *(w:w)* and incubation for 3 hours at 37 °C.

High-performance liquid chromatography

Chromatographic separation of 1 µg digested mAb was carried out on a Thermo Scientific UltiMate 3000 Rapid Separation system (Thermo Fisher Scientific, Germering, Germany) at a flow rate of 100 μL/ min employing a Thermo Scientific™ Hypersil GOLD™ aQ C18 column (100 x 1.0 mm i.d., 1.9 µm particle size, 175 Å pore size, Thermo Fisher Scientific, Sunnyvale, CA, USA) operated at a temperature of 50 °C. Mobile phase A was composed of H_2O + 0.10% formic acid (FA), mobile phase B of ACN + 0.10% FA. The total runtime for one sample was 70 minutes. The separation started at 1.0% B for 2 min followed by a linear gradient of 1−5% B in 2 min, 5–10% B in 2 min, 10–35% B in 30 min, 80% B for 4 min, and 1% B for 30 min.

Mass spectrometry

Mass spectrometry was conducted on a Thermo Scientific Q Exactive benchtop quadrupole-Orbitrap mass spectrometer equipped with a Thermo Scientific Ion Max ion source with a heated electrospray ionization (HESI) probe, both from Thermo Fisher Scientific (Bremen, Germany). The source heater temperature was set to 100°C, spray voltage to 3.5 kV, sheath gas flow to 5 arbitrary units, capillary temperature to 300°C and S-lens RF level to 60.0. Each scan cycle consisted of a full scan at a scan range of *m/z* 350–2,000 and a resolution setting of 35,000, followed by 10 data-dependent HCD scans at 29 NCE at a resolution setting of 17,500. Dynamic exclusion was set to 20 seconds.

Data evaluation

Data acquisition was conducted using Thermo Scientific Chromeleon 7.2 CDS (Thermo Fisher Scientific, Germering, Germany). Data analysis was performed using the Peptide Mapping workflow in Thermo Scientific BioPharma Finder software 3.0 (Thermo Fisher Scientific, San Jose, CA, USA) with the following settings: 10 ppm mass accuracy, minimum confidence of 0.8, specificity 'medium' for trypsin protease and the following variable modifications: glycation, succinimide, oxidation, and built-in *N*-glycan library for chinese hamster ovary cell lines, maximum allowed number of variable modifications of 2 per peptide.
